# Incidence and Management of Delayed Epistaxis Following Endoscopic Skull Base Surgery

**DOI:** 10.4274/tao.2021.5918

**Published:** 2021-03-26

**Authors:** Zahide Mine Yazıcı, Ömür Günaldı, Osman Tanrıverdi, Selçuk Güneş, Filiz Gülüstan, Recep Haydar Koç, İbrahim Sayın

**Affiliations:** 1 Department of Otorhinolaryngology, University of Health Sciences Turkey, Bakırköy Dr. Sadi Konuk Training and Research Hospital, İstanbul, Turkey; 2 Department of Neurosurgery, Bakırköy Mazhar Osman Mental Health and Neurological Diseases Training and Research Hospital, İstanbul, Turkey

**Keywords:** Epistaxis, skull base, endoscopic surgical procedure, complication

## Abstract

**Objective:**

Among other complications of endoscopic skull base surgery, delayed epistaxis has not been given much importance. This report presents postoperative delayed nosebleed cases in a large number of patients who underwent an endoscopic endonasal transsphenoidal approach to the sellar region for resection of lesions.

**Methods:**

Three hundred and sixty three patients who were reached to the sellar region by endoscopic endonasal transsphenoidal route and operated was included in the study. Retrospective chart reviewing of these patients was performed. The correlation between the duration of nosebleeds, bleeding location, treatment methods and comorbidities of the patients were evaluated.

**Results:**

Ten patients (3.6%) reported delayed epistaxis in the postoperative period and were referred to the otolaryngology department. Postoperative epistaxis occurred between days 7^th^ and 33^th^ (mean 16.5) days. The treatment consisted of chemical silver nitrate cauterization in two patients, return to the operating room in three patients, nasal packing in five patients.

**Conclusion:**

Delayed postoperative epistaxis often has no obvious etiology, and intervention requires teamworking. Well-coordinated teamworking of the neurosurgeon with other specialities such as neuroradiology and otorhinolaryngology is needed to achieve better results.

## Introduction

Today, all surgical techniques are directed towards minimally invasive techniques. Since its introduction in the 1990s, endoscopic endonasal sellar and parasellar surgery has rapidly gained acceptance in otolaryngology and neurosurgery. Endoscopic-guided transsphenoidal surgery applications have been standardized by Carrau et al. (1) and Cappabianca et al. (2). In the following years, endoscopic skull base surgery (ESBS) has become more widespread and is used in many centers today.

Although many complications related to ESBS have been discussed in the literature, epistaxis is a seldom reported complication that can increase morbidity and mortality. The rate of epistaxis after ESBS ranges from 1.7%  to 10% within the first 30 days (3).

Epistaxis after ESBS shows different features compared to other etiology of epistaxis. We evaluated the patients’ nosebleeds that occurred within the first 6 weeks after discharge as delayed epistaxis. Postoperative bleeding is ideally preferred to be stopped at the same hospital of surgery, but this may not be possible in the late postoperative bleeding. Different points of late postoperative epistaxis were tried to be emphasized in this article.

In this study, we performed a retrospective analysis of delayed nasal bleeding in a group of patients who have underwent surgery for resection of sellar lesions with ESBS.

## Methods

This study was approved by the Local Ethics Committee of Dr. Sadi Konuk Training and Research Hospital (2019/318). Informed consent was obtained from all patients who met the inclusion criteria. The study was conducted with patients who underwent ESBS for intracranial pathology at the Department of Neurosurgery, Bakırköy Mazhar Osman Mental Health and Neurological Diseases Training and Research Hospital, İstanbul between March 2014 and May 2019. All endoscopic approaches were performed by the same author (Ö.G.). The endoscopic approach to skull base was described previously in the literature (4). The patients who presented to the Emergency Department (ED) with epistaxis within six weeks after discharge from the neurosurgery clinic after transnasal endoscopic surgery were evaluated as patients with delayed epistaxis and were included for the study. Patients were excluded if an expanded skull base procedure was performed or if a nasal septal Hadad flap was used during the procedure. Also, patients whose medical records could not be accessed were excluded from the study.

Expanded skull base procedures used for accessing the ventral skull base have been classified into various anatomically based modules. In this classification scheme, the modules are defined based on their location on the sagittal and coronal/parasagittal planes. The approaches to access the median skull base (along the sagittal plane) include transfrontal, transcribriform, transplanum/transtuberculum, transclival, and transodontoid. To standardize our surgical technique, in our study, we excluded the operations in which we used the parasagittal or the coronal modules and included those which we used the transsellar approach in pathologies localized to the sellar region.

Epistaxis in the initial surgery room or before discharge were considered immediate and not included in this study. Medical records were analyzed for events during admission or ED visits. Details concerning the management of epistaxis were reviewed from medical records, including topical medicinal sprays, use of hemostatic agents, nasal packing, or surgical intervention. Common risk factors for epistaxis, including hypertension and blood thinner use were also noted.

## Results

A total of 383 consecutive patients were identified. Twenty patients’ medical records could not be accessed, and these patients were excluded from the study. In total, the medical charts of 363 patients were analyzed. Among all patients, 166 of the patients in the study were male (45.8%), 197 were female (%54.2). The average subject age was 46.2 years (range: 15 to 81). Endoscopic skull base operation indications were: 330 pituitary adenomas (126 functional adenomas, 36 recurrent adenomas, 168 nonfunctional adenomas), eleven chordomas, nine pseudotumor cerebri cases, six craniopharyngiomas, five Rathke cleft cysts, and two other pathologies. Endocrinological diagnoses in functional adenomas were: 73 acromegaly, 28 Cushing’s syndrome, 11 prolactinoma, and 14 other types of cases. Ten patients reported delayed epistaxis in the postoperative period (3.6%), presenting to an otolaryngology emergency service on mean postoperative day 16.5 (range: 7–33 days). Features of delayed epistaxis patients are listed in Table 1. Among 10 patients with bleeding, seven were female, five had high blood pressure, and four were using blood thinners. None of the patients had underlying coagulopathy indicated on their clinical history or biochemical test records. All patients were cross matched for transfusion needs.

Seven of the 10 patients who presented with epistaxis had pituitary adenoma (four acromegaly, two Cushing’s syndrome, one nonfunctional adenoma). Other indications were optic nerve decompression, arachnoid cyst in the sellar region, and rhinorrhea repair in the sphenoid sinus.

Treatments included chemical silver nitrate cauterization in two patients, return to the operating room (OR) in three, and nasal packing in five patients. In the OR, decision was made to stop the bleeding and invite the neurosurgeon who performed the first skull base surgeries of these patients to the OR. The neurosurgeon attended the operation to prevent intracranial complications while the otolaryngologist intervened to stop the bleeding. None of our patients required embolization or a blood transfusion. We preferred the sphenopalatine artery (SPA) bipolar cautery for two subjects and posterior septal bipolar cautery for one subject among these three patients. We used Merocel (Medtronic Xomed Surgical Products, Jacksonville, FL, USA) nasal tampons since they are made of synthetic open-cell foam polymer that provides a tissue-compatible and less susceptible environment for *Staphylococcus aureus* than traditional nasal gauze (5). We coated the tampons with antibiotic ointment and placed on the floor of the nasal cavity. Tampons were expanded with saline. After placing the tampons, it is necessary to observe the presence of any postnasal bleeding in the pharynx. If there was no bleeding within 48 hours, we removed the tampons and inspected the source of the bleeding. Although not statistically significant, as previously mentioned in the literature, the tendency to hemorrhage was higher in acromegaly cases compared to other pituitary adenomas (6). Although we do not have objective statistical data on this subject, our clinical observation is in this direction.

## Discussion

The posterior septal artery is a branch of SPA that runs along the lower face of the sphenoid sinus and can bleed if not cauterized in sphenoid sinus surgery. Meticulous elevation of the mucosa and bipolar coagulation of the artery before the bone work can prevent bleeding in this vessel. Most of the delayed bleeding is caused by the SPA or one of its branches (7). In case of bleeding from the SPA or its branches, electrocauterization or vascular clips can be used under OR conditions.

Excessive bleeding and deficits, especially in cranial nerves may be a warning for carotid artery bleeding (3, 4, 5, 6). It is important to remember that not all internal carotid artery (ICA) injuries manifest intraoperatively. The formation of vasospasm in the ICA following skull base surgery has been reported from a few hours up to one month after surgery (8). One of the most common complications after cavernous ICA rupture is the development of pseudoaneurysm. Pseudoaneurysm is caused by a leak or hematoma in the peripheral fibrous wall of the ICA (9). Traumatic pseudoaneurysms of the ICA resulting in epistaxis usually occur within the three weeks after the initial injury (88%) (10). Pseudoaneurysm formation is a common complication after cavernous ICA trauma. Regular angiographic screening is required in all suspected patients since there is a high risk of bleeding in the postoperative period. Fortunately, there was no carotid hemorrhage or pseudoaneurysm in our series of patients, but this does not mean that it will not happen. In this study, we evaluated this issue in detail since otolaryngologists are not very familiar.

Most ICA injuries can be prevented by observing the surgical anatomy and findings of radiologic examinations. Putting in place a clear action plan for the surgical team before intervening in such injuries can save important time and improve results. The key to treatment is controlling the initial bleeding, maintaining normotension, as well as radiologic evaluation by angiography and embolization by neuroradiology (6). Despite the many technological advances, mortality in major ICA injuries is still around 17%. The overall complication rate with balloon occlusion is between 8% and 20% (11).

Most postoperative bleeding develops within two to four weeks after surgery and there is a risk of bleeding up to five to six weeks postoperatively when the middle turbinate is resected (12). Although some authors limit nose bleeding to delayed epistaxis up to 4 weeks, we accepted 6 weeks as did Zimmer and Andaluz (3). Our neurosurgery team does not prefer resecting the middle turbinate, and when they have to, they take care to indicate this point in detail in the operation note, as this information is important when preventing stump bleeding.

Once the general condition of the patient has been stabilized, adrenaline guide pads are useful for both removing the blood clots and vasoconstricting the nasal mucosa. It is important to identify both the site and cause of the bleeding, so it can be stopped, and the cause can be treated. It is important to contact the institution where the skull base operation was done or to contact the neurosurgeon who did the initial surgery. If neither is possible, at least the medical records should be evaluated in detail. Especially, if the intervention for bleeding is done at a hospital different to the one where the initial surgery was done, it is beneficial for the two teams to communicate to obviate the complications that may occur while managing the epistaxis. During surgery, before proceeding with the intervention, it is essential to understand whether there is dehiscence on the carotid artery or to know which reconstruction method was used in the skull base surgery, the sella is reconstructed with abdominal fat, septal cartilage, middle turbinate, and other reconstructive techniques.

Thompson et al. (13) found a 3% rate for postoperative epistaxis in 30 days. While they managed most of the patients with nasal packing, one patent required chemical cautery, and five patients were treated in the OR. The authors reported that bleeding patients were elderly, male, and hypertensive.

De Los Reyes et al. (14) described 551 pituitary adenoma cases that included 457 endoscopic approaches with a postoperative epistaxis rate of 3.5%. Among their patients with delayed epistaxis, mean postoperative bleeding day was 10.8 days. Of the 12 cases with delayed epistaxis, two did not require nasal packing, the remaining 10 cases were first treated with nasal packing of different types. However, this approach was unsuccessful in five cases who were then treated with embolization. They recommended this approach in delayed epistaxis cases with no obvious etiology.

Zimmer and Andaluz (3), in their 434 consecutive endoscopic transsphenoidal patient series, found 4.1% incidence rate for epistaxis in six weeks of surgery. Treatment methods were in-office cautery in seven patients, cautery at the OR in five patients, nasal packing in three patients, embolization in two patients and use of intranasal hemostatic agents in one patient. Epistaxis that shows symptoms with deficits in the cranial nerves should be intervened in OR conditions. If the site of bleeding cannot be controlled, angiography with embolization was recommended in the referred study.

One possible bias of our study is that cases were excluded if an expanded skull base procedure was performed or if a nasal septal Hadad flap was used during the procedure. This means that the incidence of our delayed epistaxis cases after skull base surgery was 3.6%.

We manage our cases with nasal packing, cautery, and surgical exploration. The most important step in our treatment algorithm is to try to find the source of bleeding with an endoscopic nasal examination. Initial attempts should aim to control the bleeding with chemical cautery or nasal tampons, but hospitalization and surgical intervention may be needed if bleeding persists. Cranial nerve deficits are important signs in skull base epistaxis and the attending physician must be vigilant about this condition. Such patients may need angiography with embolization.

## Conclusion

To achieve better outcomes in skull base surgery, well-coordinated teamworking of the neurosurgeon with other specialities (neuroradiology, otorhinolaryngology, and others who specialize in endoscopic surgery) is needed.

It is important to reach the epicrisis and contact the surgeon while intervening in the nasal bleeding of the patient who underwent skull base operation.

**Main Points**

• Delayed epistaxis after skull base surgery can be seen up to 45 days postoperatively.

• As always, all patients presenting with postoperative epistaxis should be evaluated by nasal endoscopy to determine the bleeding site.

• Particular attention should be paid to the findings of abnormal bleeding amount and cranial nerve deficits in patients undergoing skull base surgery.

## Figures and Tables

**Table 1:**
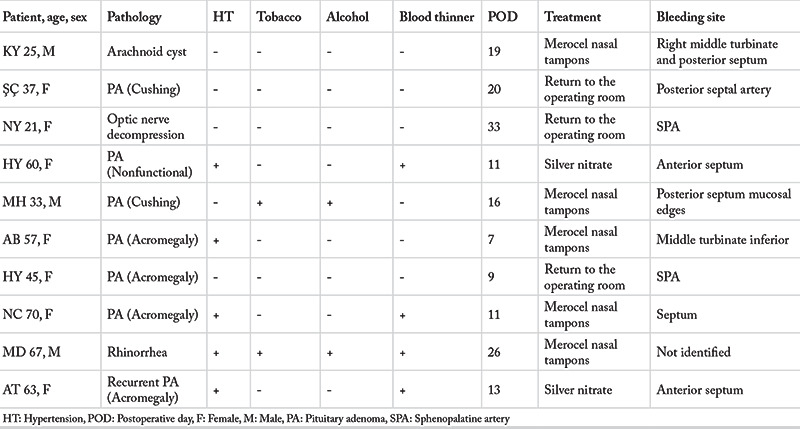
Summary of delayed epistaxis patients
